# Biodegradable Stents in the Treatment of Arterial Stenosis

**DOI:** 10.3390/jcm14020532

**Published:** 2025-01-16

**Authors:** Rasit Dinc, Evren Ekingen

**Affiliations:** 1 INVAMED Medical Innovation Institute, New York, NY 10007, USA; 2 Department of Accident and Emergency, Etlik City Hospital, Ankara 06170, Turkey; evren23@gmail.com

**Keywords:** arterial diseases, coronary artery disease, peripheral arterial disease, bioresorbable vascular stents, stent thrombosis

## Abstract

Arterial diseases (ADs) are a significant health problem, with high mortality and morbidity rates. Endovascular interventions, such as balloon angioplasty (BA), bare-metal stents (BMSs), drug-eluting stents (DESs) and drug-coated balloons (DCBs), have made significant progress in their treatments. However, the issue has not been fully resolved, with restenosis remaining a major concern. In this context, bioresorbable vascular stents (BVSs) have emerged as a promising area of investigation. This manuscript includes articles that assess the use of BVSs. Studies have identified ongoing challenges, such as negative vascular remodeling and elastic recoil post-angioplasty, stent-related injury, and in-stent restenosis following BMS placement. While DESs have mitigated these issues to a considerable extent, their durable structures are unable to prevent late stent thrombosis and delay arterial recovery. BVSs, with their lower support strength and tendency towards thicker scaffolds, increase the risk of scaffold thrombosis. Despite inconsistent study results, the superiority of BVSs over DESs has not been demonstrated in randomized trials, and DES devices continue to be the preferred choice for most cases of arterial disease. Esprit BTK (Abbott Vascular) received approval from the US FDA for below-knee lesions in 2024, offering hope for the use of BVSs in other vascular conditions. Enhancing the design and thickness of BVS scaffolds may open up new possibilities. Large-scale and longer-term comparative studies are still required. This article aims to provide an overview of the use of biodegradable stents in the endovascular treatment of vascular stenosis.

## 1. Introduction

Arterial diseases (ADs), which include coronary artery disease (CAD) and peripheral arterial disease (PAD), are a significant public health concern with high mortality and serious morbidity [1,2,3]. Angioplasty has made substantial progress in achieving vascular patency. However, restenosis remains a challenge, primarily due to negative vascular remodeling and elastic recoil after angioplasty [4]. In recent years, stents have played a crucial role in the interventional treatment of vascular occlusions [5]. While bare-metal stents (BMSs) have reduced the effects of elastic recoil and negative remodeling, they have also led to neointimal hyperplasia and eventually in-stent restenosis due to vascular response to stent-related injury [6].

To address restenosis, various endovascular intervention strategies have been developed using different platforms with varying designs, such as drug-eluting stents (DESs) and drug-coated balloons (DCBs). Despite significant progress, the issue of restenosis has not been fully resolved [7]. Additionally, the release of antiproliferative drugs from these devices has raised safety concerns, including the risk of death [8,9]. It has also been observed that durable stent structures, like stainless steel and cobalt chrome, cause chronic inflammation, disrupt reendothelialization, increase the likelihood of late stent thrombosis, and delay arterial recovery [10,11]. Moreover, these permanent stents hinder any subsequent interventions. In response to these challenges, bioresorbable vascular scaffolds (BVSs) that dissolve over time and are excreted by the body have been developed [11]. BVSs are expected to not only prevent restenosis but also avoid the long-term side effects of permanent implants [5].

The aim of this narrative review is to provide an overview of the use of biodegradable stents in the endovascular treatment of vascular stenosis.

## 2. Fundamental Characteristics of Biodegradable Stents

**Figure 1:**
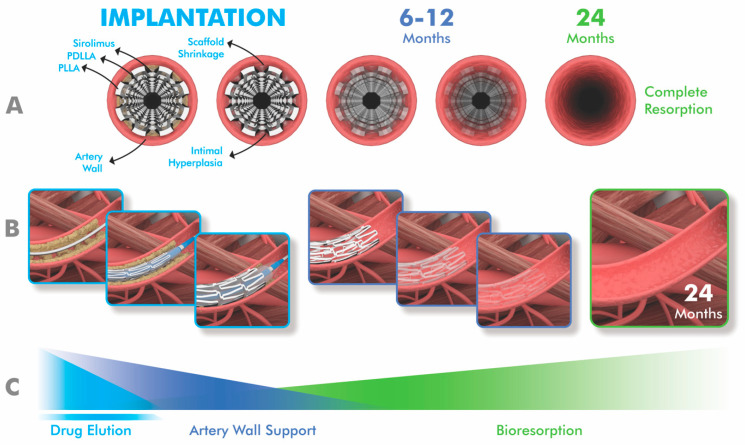
Schematic representation of the timeline of resorption of bioabsorbable vascular scaffolds and vascular reendothelization in the implanted artery from implantation to complete biodegradation. (**A**). Transverse section (**B**). Longitudinal section (**C**). Grading the drug effect, scaffold support, and bio-resorption over time. The illustrations were created using the Adobe Creative Suite Package (Photoshop, version 25.12 and Illustrator, version 28.7.1 Applications, Adobe Systems Incorporated, San Jose, CA, USA).

For a stent to be considered ideal, it should possess optimal characteristics in terms of deliverability, efficacy and safety. To elaborate further, an ideal stent should be biocompatible, flexible, deliverable and have good radial strength and fracture resistance. It should also promote vascular reendothelialization, healing and remodeling, while causing minimal inflammatory reactions and low rates of neointimal hyperplasia and stent thrombosis in long-term follow-up [12,13,14,15]. In this context, drug-eluting BVSs have attracted interest as an alternative to drug-eluting stents because they are designed to provide early mechanical support and to normalize vascular structure and function by completely resorbing over subsequent years so as to prevent very late adverse events [16,17,18,19] (Figure 1).

The most studied bioresorbable scaffold is the Absorb BVS (Abbott Vascular). The strut part consists of a balloon-expandable poly-L-lactide structure coated with a thin layer of bioresorbable poly-D L-lactide capable of releasing everolimus [20]. Various synthesized polymeric materials, such as poly-L-lactic acid (PLLA), poly(lactide-co-glycolic acid) (PLGA), poly glycolic acid (PGA), and poly-ε-caprolactone (PCL), have been widely used for the biodegradable polymeric stent struts [21]. In addition to polymers, biodegradable stent struts are also manufactured from metals such as magnesium, zinc, iron or their alloys, providing stronger radial strength and compression resistance [5]. Compared with metallic stents, those with synthetic polymer struts can be easily adapted to various structures and shapes [21]. However, they have lower radial strength than the metallic biodegradable stents [22]. On the other hand, the radial strength and compression resistance of BVSs are lower overall than those of permanent stents such as stainless steel and CoCr alloy [5,23]. Table 1 outlines the basic features of some selected commercial biodegradable stents.

Bioresorbable stents (BRSs) primarily address the need to overcome the potential limitations of DES application, such as permanent vascular caging, vasomotion limitation, adaptive vascular remodeling, and very late stent thrombosis [24]. One of the most significant drawbacks of BVS is the stent thickness, which is meant to compensate for decreased tensile and radial strength [23]. The use of thicker stents can lead to placement difficulties, especially in small-diameter vessels, as well as thrombosis and other clinical issues. Other concerns, such as polymer and scaffold degradation, have also been linked to stent thrombosis [20,25]. Among all BVSs, iron bioresorbable scaffolds (i.e., IBS, LifeTech Scientific) have the thinnest scaffold thickness, with similar support strength to other metallic scaffolds [23]. Additionally, they are expected to reduce shear stress and thrombosis due to improved reendothelialization [20]. These stents should be easily monitored with imaging methods for proper placement [23].

The PLLA-based Absorb BVS and the magnesium-based DREAMS 2G are the most representative BRS devices and have similar scaffold thicknesses [24]. Both products have been successful in humans for up to 2 years in terms of efficacy, with low rates of adverse events, primarily target lesion failure (TLF), and restoration of vasomotion and late lumen expansion [26]. Unfortunately, increased scaffold thrombosis, higher late lumen loss, and an increased risk of target lesion revascularization have limited the use of BVSs in long-term follow-up [5,17]. Although rare, adverse effects such as aneurysm and late acquisition malapposition may also be observed [27]. In recent porcine models by Gao et al., iron-based BVS struts remained intact at 6 months, and corrosion was detected at 9 months. Five-year results demonstrated efficacy and safety comparable to contemporary metal-based DES without iron artifact, intra-scaffold restenosis or thrombosis, lumen collapse, aneurysm formation, and chronic inflammation [24].

Absorb BVS (Abbott Vascular) was approved by the US Food and Drug Administration (FDA) in 2016. However, it was withdrawn in 2017 after scaffold thrombosis was revealed in several trials and meta-analyses and it is no longer commercially available anywhere [28]. Although there are currently several commercial BVS products with European Conformity (CE) approval, there is no FDA approved BVS for coronary arteries [20,29]. It is noteworthy that Esprit BTK (Abbott Vascular) was approved by the FDA in 2024 [30].

## 3. Biodegradable Stents in Coronary Arteries

BVSs were originally developed with the idea of restoring vasomotor function and reducing the risk of device thrombosis in permanent DESs. To achieve this goal, these products have been designed by combining the advantages of ensuring the effectiveness of drug-eluting stents during implantation while not leaving a foreign body behind [19]. In this concept, BVSs initially function in terms of drug-eluting and supporting the vascular wall similarly to DESs, then dissolving months to years after implantation, which in turn may lead to the restoration of vasomotor function. Once dissolved, they allow the artery to maintain its integrity and return to its physiological properties [17,18,29].

Although early reports have suggested that BVSs are superior to DESs, results from larger populations and longer-term studies have raised concerns, primarily regarding the increased risk of thrombosis, as well as major adverse cardiac events and lower radial power [5,17,19].

The first data on BVS performance are from the ABSORB I study. The 5-year data from cohorts A and B of this study were reported to be promising, with no scaffold thrombosis (ScT) observed and the rate of major adverse cardiac events (MACEs) (non-fatal stroke, non-fatal myocardial infarction, and cardiovascular death) being 3.4% and 11%, respectively [31,32]. However, the ABSORB II study, a non-inferiority study for ABSORB BVS, showed that it was associated with a two-fold increased risk of TLF compared with Xience V (which is a DES, Abbott Vascular) at 3-year follow-up (10% vs. 5%; *p* = 0.0425) [30]. The 3-year data from the ABSORB III study also show that ABSORB BVS was inferior to DES in terms of overall ST (2.4% vs. 0.6%) and TLF (11.7% vs. 8.1%) [33,34]. However, there were decreases in TLF and ScT, particularly between 3 and 5 years, compared with the 0-to-3-year time period (hazard ratios 0.83 vs. 1.35 for TLF and 0.26 vs. 3.23 for ScT, respectively). This coincides with complete scaffold resorption [35]. The 30-day results of the ABSORB IV study revealed a lower acute device success rate (94.6% vs. 99.0%), higher risk of TLF (5.0% vs. 3.7%), and a higher ischemia-induced target vessel revascularization (ID-TVR) rate (1.2% vs. 0.2%) [36]. In the 5-year follow-up data from the Absorb IV study, there was no significant difference in the rates of target vessel failure (TVF) and MACE between patients treated with BVS and those treated with CoCr-EES; there was also no significant difference in the rates of device thrombosis at 5 years after BVS and CoCr-EES (1.7% events vs. 1.1% events). The 5-year rates of TLF were reported to remain higher with BVS than with CoCr-EES (17.5% vs. 14.5%, *p* = 0.03), increasing slightly from 3-year follow-up [37].

In a large-scale study with a 12-month follow-up between durable polymer drug-eluting stents (DP-DES) and biodegradable polymer drug-eluting stents (BP-DES) groups in patients with acute myocardial infarction complicated by cardiogenic shock, Jang et al. [11] found no significant difference in clinical outcomes (1.3% vs. 1.6% for ScT and 34.2% vs. 28.5% for TVF, respectively). They also noted that they did not observe any effect of polymer technology on clinical outcomes. In patients with acute myocardial infarction, Iglesias et al. [38] have reported that the primary composite endpoint of TLF was much higher in the DP-DES group than in the BP-DES group in 12-month follow-up (6% vs. 4%: absolute risk difference −1.6%). During this period, the rates of cardiac death, target vessel myocardial reinfarction indicating clinically indicated target lesion revascularization, and definite stent thrombosis were similar between the two treatment groups. What is noteworthy in their study is that the frequency of target lesion revascularization (TLR) was much higher in the DP-DES group, emphasizing the benefits of a complete polymer degradation that reduces thrombogenicity and facilitates reendothelialization. Kim et al. [39] have reported that the risk of patient-oriented clinical outcomes was similar between the DP-DES and BP-DES groups (5.2% vs. 6.4%: absolute risk difference −1.2%), with excellent safety and efficacy profiles at 12 months for both groups. However, when the risk of device-driven clinical outcomes was assessed, there was a slight increase in the incidence of TLR in the BP-DES group (2.6% vs. 3.9%: hazard ratio, 0.67). The researchers attributed this to the properties of BP-DESs to resemble bare metal stents after polymer degradation, thus increasing the risk of late restenosis. The EVERBIO-2 trial comparing BVS and DES revealed that rates of clinical device-oriented composite events (DOCE) (29% vs. 28%, respectively) and patient-oriented composite events (POCE) (49% vs. 55%, respectively, *p* = 0.43) were not statistically significant between two groups at 10-year follow-up. On the other hand, DES was superior for some individual outcomes. For instance, the rate of target vessel myocardial infarction was 5% in the BVS group and 0% in the DES group, and the rate of possible stent thrombosis was 3% and 0% in the BVS and DES groups, respectively [17].

## 4. Biodegradable Stents in Peripheral Arteries

Stents play an important role in interventional therapy not only in cardiovascular diseases but also in peripheral arterial diseases, which affect more than 230 million people [36]. The off-label use of coronary drug-eluting stents in PAD has been a glimmer of hope [5]. However, in below-knee lesions, BMSs have failed to prove their superiority over angioplasty. DES has been successful only in short lesions (<40 mm), and usually two or more stents need to be used [40,41]. Additionally, permanent stents have been found to result in higher rates of in-stent restenosis (ISR), making them harder to re-channelize or re-dilate [42].

Dia et al. [43] evaluated the Absorb bioabsorbable vascular construct in a study of 31 patients with predominantly complex infra-popliteal lesions for the management of chronic limb ischemia with a 2-year clinical follow-up. The study reported no scaffold thrombosis or periprocedural bleeding, 100% procedural success, and 93.5% of patients were free of target vessel insufficiency at 24 months. One patient underwent revascularization and one amputation. Primary patency was 96.7% at 12 months and 87.1% at 24 months, with no deaths. Ipema et al. [43] performed a meta-analysis of first-generation drug-eluting absorbable scaffolds in peripheral vascular disease. They found that, based on 12-month evaluation, the primary patency rate was 90% (95% confidence interval (CI) 0.84–0.95), the target lesion revascularization (TLR) rate was 96% (95% CI 0.91–0.99), and the limb salvage rate was 97% (95% CI 0.91–0.99). 

**Figure 2:**
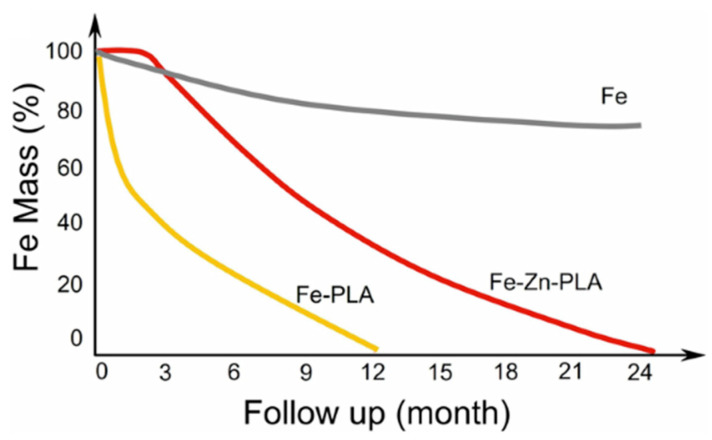
Schematic illustration of the effect of PLA on the degradation profile of nitrided iron (partially reprinted from [5]). Fe, iron; PLA: poly-lactic acid; Zn, zinc.

First-generation drug-eluting absorbable scaffolds were withdrawn from the market due to the increase in target vessel myocardial infarction after CAD use. However, they still have potential for application in the treatment of PAD [44]. It should be pointed out that, although polymer-based BVSs have been found to be sufficient for the patency of short lesions in the infrapopliteal artery, their use has been limited due to their shorter length and larger support thickness [45]. Although iron-based BVSs show better radial strength, pure iron generally has significant disadvantages, such as slow corrosion and bio-resorption [46]. Zhang et al. [5] have used long biodegradable sirolimus-eluting stents (LBSs) with nitrided and polylactic acid (PLA)-coated iron in below-the-knee (BTK) lesions, which have a higher fragility than other lower extremity vascular segments. They have reported that LBSs with 70 μm support thickness and lengths up to 118 mm were safe and feasible according to 13-month follow-up results. They have also reported that proper iron nitriding improved the mechanical performance of the metal, while PLA coating accelerated its degradation (Figure 2) [5,46]. 

The pivotal investigation of safety and efficacy of drug-eluting resorbable scaffold treatment—below the knee (LIFE-BTK) trial, is a randomized controlled trial (RCT) designed to prospectively evaluate the premarket evaluation of the Esprit BTK drug-eluting absorbable scaffold (Abbott Vascular) for the treatment of patients with chronic limb-threatening ischemia (CLTI) and infrapopliteal artery disease. Initial results have revealed that the LIFE-BTK is superior to angioplasty in the primary efficacy endpoint for BTK lesions (74% vs. 44%: 95% CI, *p* < 0.001) at 1-year follow-up. Additionally, the primary safety endpoint (freedom from major adverse limb events and perioperative death at 6 months) was non-inferior to angioplasty (with an absolute difference of –3 percentage points: 95% CI, *p* < 0.001) [40]. In 2024, Esprit BTK was approved by the FDA for BTK, and currently, it is the only BVS approved by the FDA worldwide [28]. This approval perhaps keeps the hope of the bioresorbable scaffold concept alive.

## 5. Future Perspective

Although BVSs have various advantages, they also face some difficulties. First, they exhibit insufficient mechanical strength. Second, the degradation time of the stent is not always compatible with that of vascular remodeling. Finally, the implantation method presents challenges for some diseased areas [21]. 

Good mechanical properties and stent stability until intima formation in the neovascularization wall are sought in an ideal stent. However, current BVSs generally have disadvantages of stent thickness and scaffold disintegration, leading to stent thrombosis [47]. Therefore, BVSs need to have mechanical properties that perform at least as well as DES in the short term and better than DES in the long term. Additionally, the use of technologies such as 3D printing as a manufacturing technique opens the door to patient-specific devices that can meet the precise requirements of each individual. When these ideal characteristics are achieved, challenges such as immunogenicity, inflammation, fibrous tissue formation, material degradation, and cytotoxicity can be addressed more easily.

Stents that can locally deliver anti-coagulant or anti-inflammatory drugs will provide significant benefits in terms of greater efficacy and reduced off-target effects. The stent should also be visible for monitoring during and after the intervention. Finally, smart stents that are easy to deploy and adapt well to the target could prevent restenosis while simultaneously offering the ability to monitor post-implantation outcomes.

## 6. Discussion and Conclusions

Although the comparison of DP-DES with BP-DES showed contradictory results, DP-DES was not, overall, inferior to BP-DES in terms of TVF, and no additional clinical benefit was observed from BP-DES compared with DP-DES during the follow-up periods [48]. On the other hand, in some studies, the high adverse event rates of BVSs have been attributed to the inadequate mechanical properties of the first-generation scaffold and the inadequate implantation technique. Bengueddache et al. [17] analyzed the EverBio-2 study over a 10-year follow-up, which was the first published evaluation over 10 years on BVS. They noted that the initial analysis showed comparable clinical outcomes for DESs and BVSs at 9 months, followed by subsequent studies showing no significant difference in clinical outcomes at 2 and 5 years. They also found that, over 10 years, DOCE and POCE rates were similar between the BVS and DES groups. When looking at individual adverse events, possible stent thrombosis was found to be higher in the BVS group after 10 years. These researchers also evaluated the results of other notable studies on BVS, such as ABSORB-JAPAN, ABSORB-III, and ABSORB-IV. In these studies, the 5-year stent thrombosis rate ranged from 1% to 3.8% in the BVS groups. This rate was significantly higher than in the DES group only in the ABSORB-III study comparing the Everolimus eluting stent group (BVS = 2.5%, EES = 1.1%; *p* = 0.03) [17].

To achieve the best clinical outcome, an aggressive lesion preparation and postdilation strategy is important during scaffold delivery with intracoronary imaging [49]. IVUS is well known for detecting suboptimal stent outcomes and improving the outcome of PCI without any safety concerns. The use of IVUS during percutaneous interventions results in significantly lower rates of adverse events such as target vessel failure and stent thrombosis [50]. The monitoring of BRS with optical coherence tomography (OCT) provides important information regarding the percentage of covered supports, the extent of neointima coverage, and support removal. These assessments also provide information on the long-term performance of BRS [51].

After stent placement, direct exposure of the subendothelial layer containing collagen and tissue factors to blood causes activation of platelets and the coagulation system [21]. This may eventually lead to stent thrombosis. However, there are no data indicating the optimal duration of dual antiplatelet therapy (DAPT) after BRS implantation or that indicate that this duration can be shortened to 3 months [20]. Therefore, aggressive antiplatelet therapy is recommended in biodegradable stenting [49].

It has been suggested that the situation would improve with enhancements in these features [16,37]. In this regard, the development of thinner struts, improved placement technique and the ideal period of disintegration in the human body (especially for iron scaffolds) may reduce shear stress and thrombosis, resulting in improved reendothelialization [5,20].

Based on current experience, it is not recommended to implant BVSs in vessels with reference vessel diameters <2.25 mm and >3.75 mm. This is because BVSs tend to have thicker and wider scaffolds, increasing the risk of ScT [23]. Although iron-based scaffolds are thinner and more durable, they take longer to corrode completely, up to 5–6 years [46].

When comparing the advantages and disadvantages of DES and BVS, DES offers benefits in terms of stent thrombosis, strut thickness and dismantling. On the other hand, DESs have potential drawbacks compared with BVSs in terms of late and very late stent thrombosis, very late strut fracture and neoatherosclerosis [20].

The strut thickness of Absorb (>150 μm) has been shown to result in greater luminal protrusion and turbulent flow, delayed reendothelialization, and increased neointimal hyperplasia. Thinner BVS scaffolds with superior mechanical performance are expected to offer potential improvement [16]. Firesorb (MicroPort) BRS is a thinner-supported (100–125 μm) PLLA-based sirolimus-eluting BRS designed to reduce luminal protrusion and enhance blood flow dynamics. This product has demonstrated nearly the same 1-year angiographic intra-segment late loss and tissue strut coverage as CoCr-EES without scaffold thrombosis in the FUTURE-II randomized trial [52]. Esprit, a PLLA-based scaffold, has a support thickness of 99–120 μm and has been approved by the FDA for BTK based on favorable results. It also has four platinum markers, two of the same mass, embedded in the proximal and distal ends of the scaffold for radiopacity [44]. Preclinical evaluations of the iron-based BVS scaffold with a support thickness of 70 μm showed promising results [24,53]. It is recommended that BRS be implanted under intravascular imaging guidance, as BRS failure has been largely attributed to suboptimal implantation technique in addition to thick strut [20]. Ali et al. and Cassese et al. conducted two different meta-analysis studies comparing BVS and DES. Ali et al. [54] found that BVS resulted in higher 3-year TLF rates compared with CoCr-EES (11.7% vs. 8.1%; RR, 1.38; 95% CI, 1.10–1.73; *p* = 0.006). Similarly, results from the study by Cassese et al. [55] showed a higher risk of TLF in BVS compared with EES (OR, 1.35; 95% CI, 1.11–1.65; *p* = 0.0028). The higher rates in the BVS group were mainly due to target vessel myocardial infarction in both groups. Both studies also reported higher rates of ScT in the BVS group. In contrast, Lu et al.’s [56] meta-analysis with 5-year follow-up found that BP-BES was associated with lower rates of TLR (OR, 0.77; 95% CI, 0.62–0.96), ScT (OR, 0.60; 95% CI, 0.43–0.84) and MACE (OR, 0.83; 95% CI, 0.71–0.97). In a 12-month follow-up evaluation of BVS implantation in acute coronary syndrome (ACS), Rzeszutko et al. [49] reported a DOCE rate of 3.2% and a MACE rate of 4.44%. Based on their observations during this period, they stated that BVS can be used successfully and safely in patients with ACS and complex lesions including calcified lesions and severely tortuous vessels.

BRSs have the potential to restore natural vasomotion by gradually dissolving. This restoration is more important for peripheral arteries. Although direct studies on vasomotion of BRSs are limited, current research indicates that they will contribute to the artery returning to its natural state [44,57].

In conclusion, the superiority of the BVS over DES has not been demonstrated in a randomized trial, and DES devices are still the first choice for most arterial disease cases. These results have drawn significant attention to the safety of BTSs, leading to a decrease in the initial appeal of BVSs. Late scaffold thrombosis is the main adverse event and the ongoing limitation of their preference. The FDA’s approval of Esprit BTK for use in below-knee lesions may be a promising light for the use of BVSs in other vascular events.

The decrease in scaffold thrombosis rates, observed simultaneously with the complete resorption of BVS and the reduction in the risk of early BVS that is achieved by improving the scaffold design and placement technique, may be important guiding data to produce refined new BVSs. To further elucidate the usability of BVSs, future large-scale and longer-follow-up comparative studies are needed to assess their effectiveness and safety.

## Figures and Tables

**Table 1:** Some selected commercial biodegradable polymeric and metallic scaffolds [16,23,24].

Device (Manufacturer)	Scaffold	Coating	Anti-Proliferative Drug	Strut Thickness (μm)	Bio-Resorption Time	Note
Biodegradable polymeric scaffold						
Absorb BVS (Abbott Vascular, Santa Clara, CA, USA)	PLLA	PDLA	Everolimus	157	24–48	No longer commercially available
DESolve Cx (Elixir Medical, Milpitas, CA, USA)	PLLA	Polylactide-based polymer	Novalimus	150	24–36	CE marked
ART Pure (Arterial Remodeling Technologies, Paris, France)	PDLLA	None	Drug-free	170	12–24	CE marked
MeRes100 (Meril Life Sciences, Gujarat, India)	PLLA	PDLLA	Sirolimus	100	24	CE marked
Fantom (Reva Medical, San Diego, CA, USA)	PTD-PC	PTD-PC	Sirolimus	125	36	CE marked
Xinsorb (Huaan Biotechnology, Jinan, China) *	PLLA	PDLLA	Sirolimus	160	24–36	
Biodegradable metal scaffold						
Magmaris (Biotronik, Bülach, Switzerland)	Magnesium alloy	PLLA	Sirolimus	150	12	CE marked
IBS (LifeTech Scientific, Shenzhen, China) *	Iron alloy	PDLLA	Sirolimus	70	12–24	
Below-the-knee						
Esprit BTK (Abbott Vascular, Santa Clara, CA, USA)	PLLA	PDLLA	Everolimus	<100 ⇒It is indicated for the improvement of lumen diameter in infra-popliteal lesions in patients with CLTI with scaffold length up to 170 mm and reference vessel diameter of 2.5–4.00 mm.		FDA approved

* Not CE-approved. Abbreviations: PLLA, poly-l-lactide; PDLLA, poly-d, l-lactide; NA, PTD-PC, polymer of desaminotyrosine polycarbonate; CLTI, chronic limb-threatening ischemia.

## Data Availability

Not applicable.
